# MRAP2 inhibits β-arrestin recruitment to the ghrelin receptor by preventing GHSR1a phosphorylation

**DOI:** 10.1016/j.jbc.2022.102057

**Published:** 2022-05-20

**Authors:** Alix A.J. Rouault, Paul Buscaglia, Julien A. Sebag

**Affiliations:** 1 Department of Molecular Physiology and Biophysics, University of Iowa, Carver College of Medicine, Iowa City, Iowa, USA; 2 F.O.E.D.R.C, Iowa City, Iowa, USA; 3 Pappajohn Biomedical Institute, Iowa City, Iowa, USA; 4 Iowa Neuroscience Institute, Iowa City, Iowa, USA

**Keywords:** CHO, Chinese hamster ovary, GHSR1a, growth hormone secretagogue receptor 1a, GPCR, G-protein–coupled receptor, GRK2, GPCR kinase 2, HA, hemagglutinin, ICL3, third intracellular loop, IP, inositol triphosphate, PBST, PBS with Tween 20

## Abstract

The melanocortin receptor accessory protein 2 (MRAP2) is essential for several physiological functions of the ghrelin receptor growth hormone secretagogue receptor 1a (GHSR1a), including increasing appetite and suppressing insulin secretion. In the absence of MRAP2, GHSR1a displays high constitutive activity and a weak G-protein–mediated response to ghrelin and readily recruits β-arrestin. In the presence of MRAP2, however, G-protein–mediated signaling *via* GHSR1a is strongly dependent on ghrelin stimulation and the recruitment of β-arrestin is significantly diminished. To better understand how MRAP2 modifies GHSR1a signaling, here we investigated the role of several phosphorylation sites within the C-terminal tail and third intracellular loop of GHSR1a, as well as the mechanism behind MRAP2-mediated inhibition of β-arrestin recruitment. We show that Ser^252^ and Thr^261^ in the third intracellular loop of GHSR1a contribute to β-arrestin recruitment, whereas the C-terminal region is not essential for β-arrestin interaction. Additionally, we found that MRAP2 inhibits GHSR1a phosphorylation by blocking the interaction of GRK2 and PKC with the receptor. Taken together, these data suggest that MRAP2 alters GHSR1a signaling by directly impacting the phosphorylation state of the receptor and that the C-terminal tail of GHSR1a prevents rather than contribute to β-arrestin recruitment.

The “hunger hormone” ghrelin is secreted by X/A cells of the oxyntic mucosa of the stomach in response to a low energetic state, which leads to an increase in appetite (1, 2) and prevents hypoglycemia (3, 4). Ghrelin is the agonist of the growth hormone secretagogue receptor 1a (GHSR1a), a G-protein–coupled receptor (GPCR) expressed in the brain and in multiple peripheral organs including the heart and the endocrine pancreas. Activation of GHSR1a by ghrelin in hypothalamic agouti-related protein (AgRP) neurons potently stimulates feeding (5, 6, 7). In pituitary somatotrophs, GHSR1a stimulation promotes growth hormone release (8, 9, 10). Finally, in cardiomyocytes, ghrelin increases cell survival and contractility (11, 12) while in the endocrine pancreas the hormone inhibits insulin secretion (13, 14).

GHSR1a primarily couples to Gα_q/11_, thus stimulating the production of intracellular inositol triphosphate (IP) 3. Like other GPCRs, agonist stimulation results in phosphorylation of GHSR1a by kinases, including GPCR kinase 2 (GRK2) and PKC (15), and β-arrestin recruitment. Notably, GHSR1a contains several phosphorylation sites within the C-terminal tail, some of which have been shown to be important for β-arrestin recruitment (16). However, although other putative phosphorylation sites are present in the third intracellular loop (ICL3) of GHSR1a, their role in β-arrestin recruitment has not yet been described.

When expressed in heterologous cells, GHSR1a displays a high constitutive activity and a limited ghrelin-stimulated responses (17).Both constitutive- and agonist-stimulated GHSR1a signaling are regulated by the single transmembrane melanocortin receptor accessory protein 2 (MRAP2), which functions to drastically reduce GHSR1a constitutive activity and increase ghrelin-stimulated responses (17). Additionally, MRAP2 significantly inhibits ghrelin-induced β-arrestin recruitment to GHSR1a (17). As such, MRAP2 is essential for several physiological functions of ghrelin including its orexigenic activity (18) and its insulinostatic actions (14). Global or AGRP neuron–targeted deletion of MRAP2 abrogates the effect of ghrelin on food intake (18) and global or pancreatic δ-cell-targeted deletion of MRAP2 prevents ghrelin-mediated inhibition of insulin secretion (14).

Although expressed in AGRP neurons and pancreatic δ-cells (thus promoting G-protein coupling and inhibiting β-arrestin-dependent signaling), MRAP2 is not present in every GHSR1a-expressing tissue. Consequently, it is possible that β-arrestin signaling plays an important role in the physiological function of ghrelin in tissues where MRAP2 is absent. Whereas, the inhibition of β-arrestin recruitment to GHSR1a by MRAP2 is well established and the domains of MRAP2 required for this function have been identified (17), the molecular mechanism by which MRAP2 alters GHSR1a signaling is not yet understood. In this study, we investigated the importance of GHSR1a phosphorylation for β-arrestin recruitment and the mechanism involved in MRAP2-mediated inhibition of β-arrestin recruitment.

## Results

### Deletion of the C-terminal tail of GHSR1a does not prevent β-arrestin recruitment

**Figure 1:**
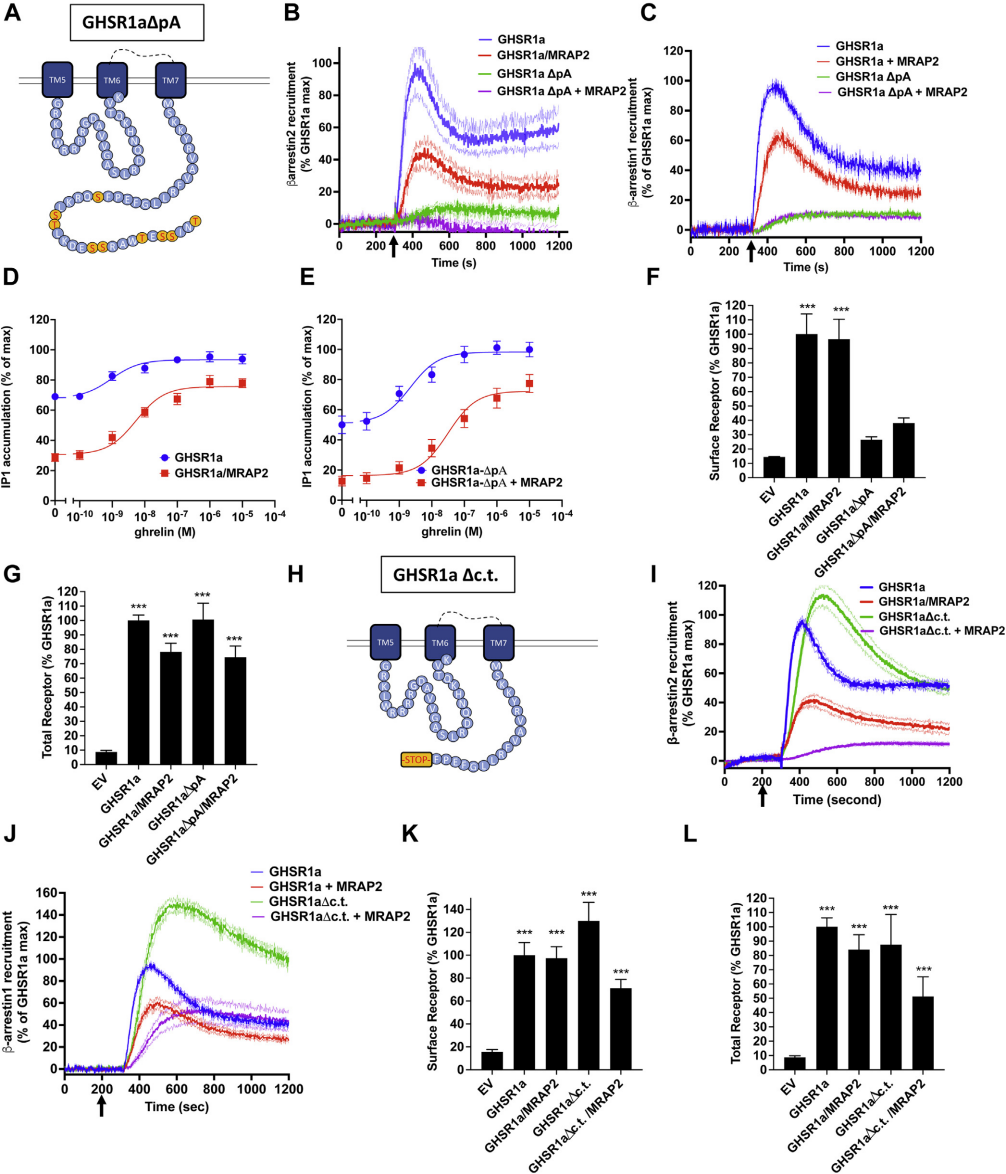
**Role of the C-terminal tail of GHSR1a β-arrestin recruitment.***A*, schematic representation of the ICL3 and C-terminal region of GHSR1a highlighting the phosphorylation sites mutated to alanine in the GHSR1aΔpA mutant. *B*, ghrelin stimulated β-arrestin2 recruitment to GHSR1a and GHSR1aΔpA with and without MRAP2. *Arrow* indicates ghrelin injection time. *C*, ghrelin stimulated β-arrestin1 recruitment to GHSR1a and GHSR1aΔpA with and without MRAP2. *D*, ghrelin-stimulated IP1 accumulation assay in cells transfected with GHSR1a with and without MRAP2. *E*, ghrelin-stimulated IP1 accumulation assay in cells transfected with GHSR1aΔpA with and without MRAP2. *F*, detection of GHSR1a and GHSR1aΔpA with and without MRAP2 by ELISA in nonpermeabilized cells. *G*, detection of GHSR1a and GHSR1aΔpA with and without MRAP2 by ELISA in permeabilized cells. *H*, schematic representation of the ICL3 and C-terminal region of GHSR1aΔc.t. *I*, ghrelin stimulated β-arrestin2 recruitment to GHSR1a and GHSR1aΔc.t with and without MRAP2. *J*, ghrelin stimulated β-arrestin1 recruitment to GHSR1a and GHSR1aΔc.t with and without MRAP2. *K*, detection of GHSR1a and GHSR1aΔc.t with and without MRAP2 by ELISA in nonpermeabilized cells. *L*, detection of GHSR1a and GHSR1aΔc.t with and without MRAP2 by ELISA in permeabilized cells. Data are the mean ± SEM of three independent experiments performed in triplicate. ∗∗∗*p* < 0.001. GHSR1a, growth hormone secretagogue receptor 1a; ICL3, third intracellular loop.

The C-terminal tail of GHSR1a contains nine putative phosphorylation sites, several of which, specifically Ser^362^, Ser^363^, and Thr^366^, have previously been shown to contribute to β-arrestin recruitment (16). To test the importance of phosphorylation of the C-terminal tail in modulation of β-arrestin recruitment, we generated a mutant GHSR1a in which all serines (344, 349, 355, 356, 362, and 363) and threonines (350, 360, and 366) within the C-terminal region were substituted with alanines (GHSR1aΔp-A)(Fig. 1*A*). β-arrestin recruitment was measured using the previously described NanoBiT recombination assay (17) with LgBiT fused to the C terminus of 3hemagglutinin (HA)-GHSR1a and SmBiT fused to the N terminus of β-arrestin.

In Chinese hamster ovary (CHO) cells expressing WT receptor, ghrelin triggered a rapid and readily measurable recruitment of β-arrestin2; as previously shown, coexpression of MRAP2 blunted this effect (Fig. 1*B*, (17)). In contrast, virtually no measurable β-arrestin2 recruitment was recorded following stimulation of GHSR1aΔp-A with ghrelin regardless of MRAP2 expression (Fig. 1*B*). Similar results were obtained after testing β-arrestin1 recruitment to GHSR1a and GHSR1aΔp-A (Fig. 1*C*), although MRAP2 only decreased β-arrestin1 recruitment by 40%. To test that the GHSR1aΔp-A mutant was functional, we measured ghrelin-stimulated IP1 accumulation in cells transfected with either GHSR1a or GHSR1aΔp-A alone or in the presence of MRAP2. Similar to WT GHSR1a (Fig. 1*D*, (17)), GHSR1aΔp-A receptors displayed high constitutive activity in the absence of MRAP2 and MRAP2 expression reduced basal receptor-mediated activity while increasing the amplitude of the ghrelin-stimulated response, suggesting that mutations of the C-terminal phosphorylation sites do not impair signaling (Fig. 1*E*).

We then measured the surface density of 3HA-GHSR1a-LgBiT and 3HA-GHSR1aΔp-A-LgBiT cotransfected with SmBiT-β-arrestin using ELISA in nonpermeabilized cells. Whereas, GHSR1aΔp-A signaling was similar to the WT receptor, the surface density of GHSR1aΔp-A was greatly reduced (Fig. 1*F*) with no significant difference in the total expression measured in permeabilized cells (Fig. 1*G*), suggesting that mutations in GHSR1aΔp-A result in impaired trafficking to the plasma membrane. This finding complicates the interpretation of the β-arrestin recruitment assay since the decrease in receptor surface density could explain the loss of β-arrestin recruitment. Therefore, to further assess the importance of the distal C-terminal tail of GHSR1a and its nine putative phosphorylation sites in modulation of β-arrestin recruitment, we generated a truncated form of GHSR1a by deleting the amino acids after phenylalanine 343 (Fig. 1*H*). Surprisingly, truncation of the distal C-terminal tail fragment did not impair either β-arrestin2 or β-arrestin1 recruitment (Fig. 1,*I* and *J*). In fact, β-arrestin1 recruitment was increased by 50%. However, the inhibitory effect of MRAP2 on β-arrestin recruitment was also enhanced (Fig. 1,*I* and *J*). In contrast to GHSR1aΔp-A, the surface density of GHSR1aΔc.t. was slightly higher than the WT receptor (Fig. 1*K*) with no difference in total expression (Fig. 1*L*).

Together, these experiments show that, in accordance with previous reports (16), phosphorylation of the C-terminal region of GHSR1a is important for β-arrestin recruitment to the receptor; however, since the deletion of the C-terminal domain of GHSR1a containing the phosphorylation sites does not impair interaction of β-arrestins with the receptor, our result suggest that binding of β-arrestin to GHSR1a does not require interaction with the phosphorylated C-tail. This finding suggests that, at least in the absence of the C-terminal tail of the receptor, β-arrestin can interact with other phosphorylation sites localized in intracellular loops of GHSR1a.

### Phosphorylation sites within the ICL3 of GHSR1a are important for β-arrestin recruitment

Since the deletion of the distal C-terminal tail region of GHSR1a containing the putative phosphorylation sites did not prevent β-arrestin recruitment, we tested the importance of two putative phosphorylation sites contained within the ICL3 (Ser^252^ and Thr^261^). To determine whether either residue is required to recruit β-arrestin, we generated two mutants of GHSr1a-LgBiT, in which either Ser^252^ or Thr^261^ were substituted for an alanine. LgBiT-fused receptors with either mutation were transfected in CHO cells with SmBiT-β-arrestin1 or SmBiT-β-arrestin2 in the presence or absence of MRAP2.

**Figure 2:**
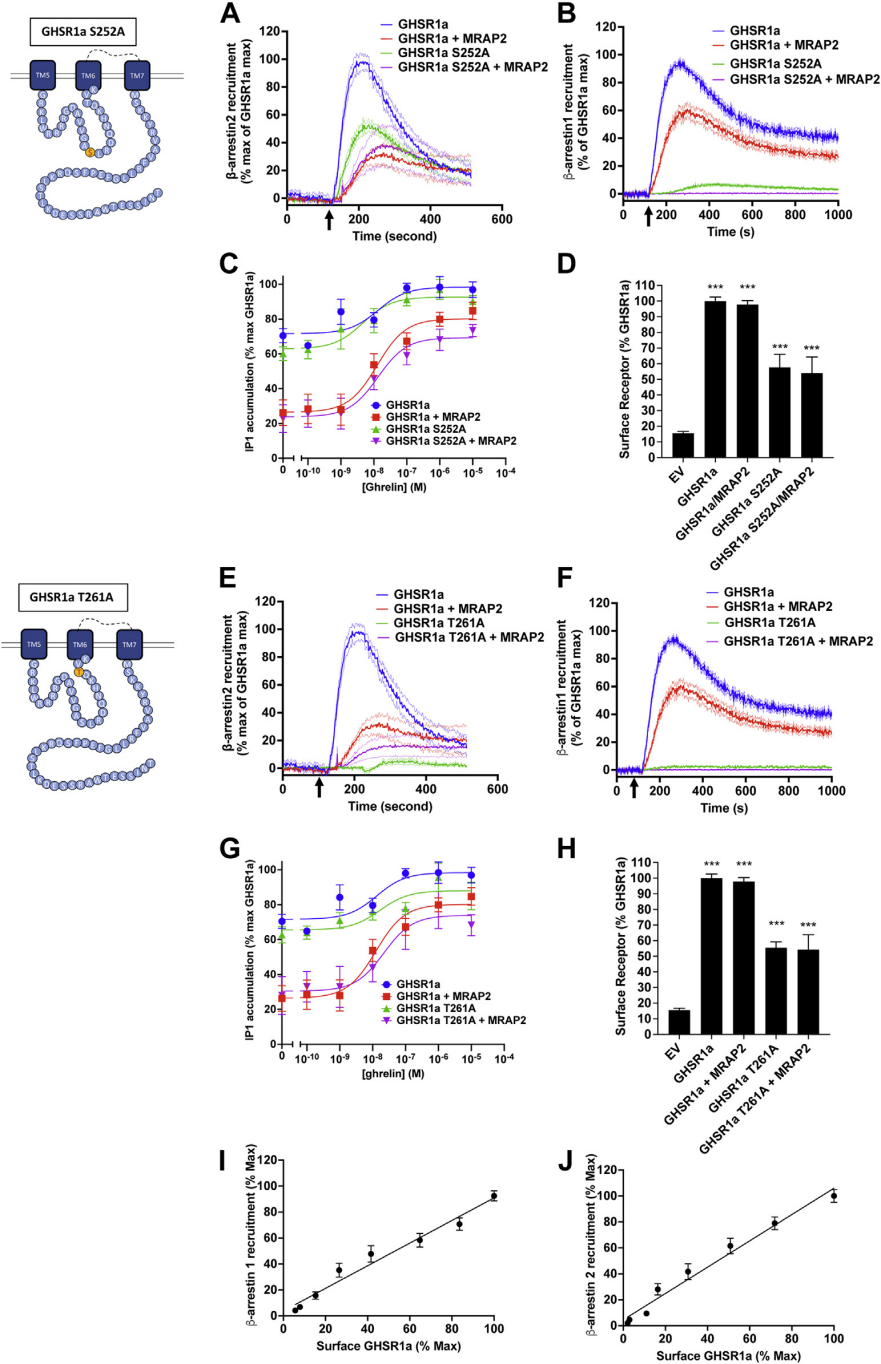
**Role of GHSR1a ICL3 residues Ser252 and Thr261 for recruitment of β-arrestin.***A*, ghrelin stimulated β-arrestin2 recruitment to GHSR1a and GHSR1a-S252A with and without MRAP2. *Arrow* indicates ghrelin injection time. *B*, ghrelin stimulated β-arrestin1 recruitment to GHSR1a and GHSR1a-S252A with and without MRAP2. *C*, ghrelin-stimulated IP1 accumulation assay in cells transfected with GHSR1a and GHSR1a-S252A with and without MRAP2. *D*, detection of GHSR1a and GHSR1a-S252A with and without MRAP2 by ELISA in nonpermeabilized cells. *E*, ghrelin stimulated β-arrestin2 recruitment to GHSR1a and GHSR1a-T261A with and without MRAP2. *F*, ghrelin stimulated β-arrestin1 recruitment to GHSR1a and GHSR1a-T261A with and without MRAP2. *G*, ghrelin-stimulated IP1 accumulation assay in cells transfected with GHSR1a and GHSR1a-S252A with and without MRAP2. *H*, detection of GHSR1a and GHSR1a-T261A with and without MRAP2 by ELISA in nonpermeabilized cells. Data are the mean ± SEM of three independent experiments performed in triplicate. *I* and *J*, ghrelin-stimulated β-arrestin1 (*I*) and b-arrestin2 (*J*) in function of surface GHSR1a expression measured by ELISA. ∗∗∗*p* < 0.001. GHSR1a, growth hormone secretagogue receptor 1a; IP, inositol triphosphate.

Mutation of Ser^252^ resulted in a 50% decrease in β-arrestin2 recruitment while β-arrestin1 recruitment was almost completely abrogated (Fig. 2, *A* and *B*). In contrast, ghrelin-stimulated IP3 production was similar between WT and GHSR1a-S252A expressing cells; coexpression with MRAP2 reduced constitutive activity and enhanced the amplitude of the ghrelin-stimulated response similarly for the WT and mutated receptor (Fig. 2*C*). Surface expression of GHSR1a-S252A was about 40% lower than GHSR1a, suggesting the presence of spare receptors since signaling was not affected (Fig. 2*D*). Interestingly, the T261A mutation largely abrogated the ability of GHSR1a to recruit both β-arrestin2 and β-arrestin1 (Fig. 2, *E* and *F*), and, like GHSR1a-S252A, signaling of GHSR1a-T261A was similar to the WT receptor while surface expression was about 40% lower (Fig. 2, *G* and *H*). To determine whether the change in surface expression of the Ser^252^ and Thr^261^ mutants could explain the decrease in β-arrestin recruitment, we transfected cells with several concentrations of the 3HA-GHSR1a plasmid and measured both ghrelin-stimulated β-arrestin recruitment and receptor surface density. We found that β-arrestin recruitment is linearly proportional to the amount of receptor expressed at the cell surface (Fig. 2,*I* and *J*); consequently, while the decrease in surface density of GHSR1a-S252A may be in part responsible for the decrease in β-arrestin2 recruitment observed for this mutant (Fig. 2*A*), the virtually complete abrogation of β-arrestin1 recruitment to GHSR1a-S252A (Fig. 2*B*) and the loss of recruitment of both β-arrestins to GHSR1a-T261A (Fig. 2, *E* and *F*) are not due to the 30% decrease in receptor density. These results suggest an important role for the two phosphorylation sites within the ICL3 of GHSR1a for β-arrestin recruitment.

### MRAP2 inhibits GHSR1a phosphorylation

**Figure 3:**
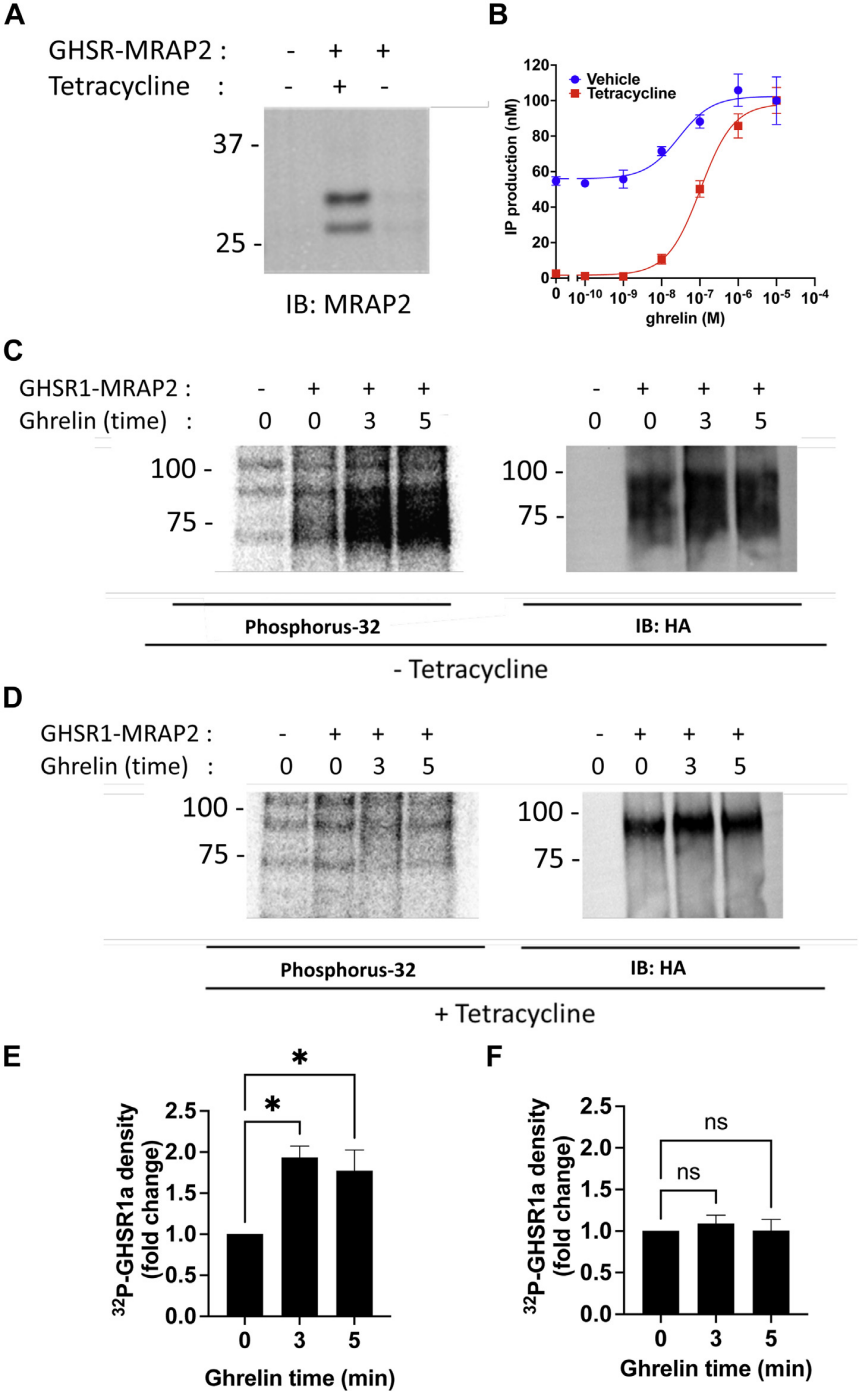
**MRAP2 inhibits the phosphorylation of GHSR1a.***A*, Western blot detection of MRAP2 in lysates of CHO-T-REx cells transfected with empty vector or with the bigenic HSVTK-3HA-GHSR1a-(CMV)/TetO2-MRAP2 construct in the presence or absence of tetracycline. *B*, ghrelin-stimulated IP1 accumulation assay in cells transfected with the bigenic construct in the presence or absence of tetracycline. *C*, ^32^P phosphoimaging and Western blot detection of GHSR1a following immunoprecipitation from cells transfected with HSVTK-3HA-GHSR1a-(CMV)/TetO2-MRAP2 without tetracycline and treated with ghrelin for the indicated time. *D*, ^32^P phosphoimaging and Western blot detection of GHSR1a following immunoprecipitation from cells transfected with HSVTK-3HA-GHSR1a-(CMV)/TetO2-MRAP2 with tetracycline and treated with ghrelin for the indicated time. *E* and *F*, densitometry analysis of GHSR1a phosphorylation in cells treated without (*E*) or with (*F*) tetracycline. Data are the mean ± SEM of three independent experiments. ∗ *p* < 0.05. CHO, Chinese hamster ovary; CMV, cytomegalovirus; GHSR1a, growth hormone secretagogue receptor 1a; IP, inositol triphosphate.

Since MRAP2 suppresses ghrelin-mediated β-arrestin recruitment to GHSR1a and phosphorylation of GPCRs is a critical step leading to β-arrestin recruitment, we next investigated whether expression of MRAP2 modified ghrelin-stimulated incorporation of phosphorus-32 (^32^P) within GHSR1a. For this, we generated a bigenic plasmid containing the coding sequence for 3HA-GHSR1a and MRAP2 driven by the HSVTK promoter and a tetracycline-inducible promoter, respectively. To test the construct, we transfected CHO-T-REx cells with mock (GFP) plasmid or the bigenic construct in the presence or absence of tetracycline. CHO-T-REx cells were used because they display very low leakiness of the tetracycline-inducible promoter. This was verified by Western blot detection of MRAP2 in lysates from cells transfected with the bigenic plasmid. We found that, using this plasmid, MRAP2 was only detectable after treatment with tetracycline (Fi. 3*A*). The activity of MRAP2 on GHSR1a in this system was verified by measuring ghrelin-stimulated IP3 production in CHO-T-Rex cells transfected with the bigenic construct in the presence and absence of tetracycline. Here again, the signaling profile of cells without or with tetracycline matched previous results using cells transfected with GHSR1a alone or with MRAP2, respectively (Fig. 3*B*). The choice of using a bigenic construct for the phosphorylation studies was made because it allows measurement of GHSR1a ^32^P incorporation with or without MRAP2 expression in cells issued from the same transfection, thereby improving the reliability and accuracy of the assay.

CHO-TREX cells were transfected with GFP or the bigenic plasmid in the presence or absence of tetracycline, incubated with [^32^P]-orthophosphate and stimulated with 1 μM ghrelin for 0, 3, or 5 min. GHSR1a expression was verified by Western blot and ^32^P incorporation was measured by radioactive phosphoimaging using a Typhoon imager. As expected, ghrelin stimulation resulted in rapid phosphorylation of GHSR1a (Fig. 3*C*); however, in cells treated with tetracycline (thus expressing MRAP2), no phosphorylation of the receptor was detectable following ghrelin treatment (Fig. 3*D*). Phosphorylation changes in both conditions were quantified by densitometry analysis (Fig. 3, *E* and *F*). These results establish that MRAP2 prevents ghrelin-stimulated GHSR1a phosphorylation, which provides a mechanism for the previously observed inhibition of β-arrestin recruitment by MRAP2.

### MRAP2 inhibits the interaction of GRK2 with GHSR1a

**Figure 4:**
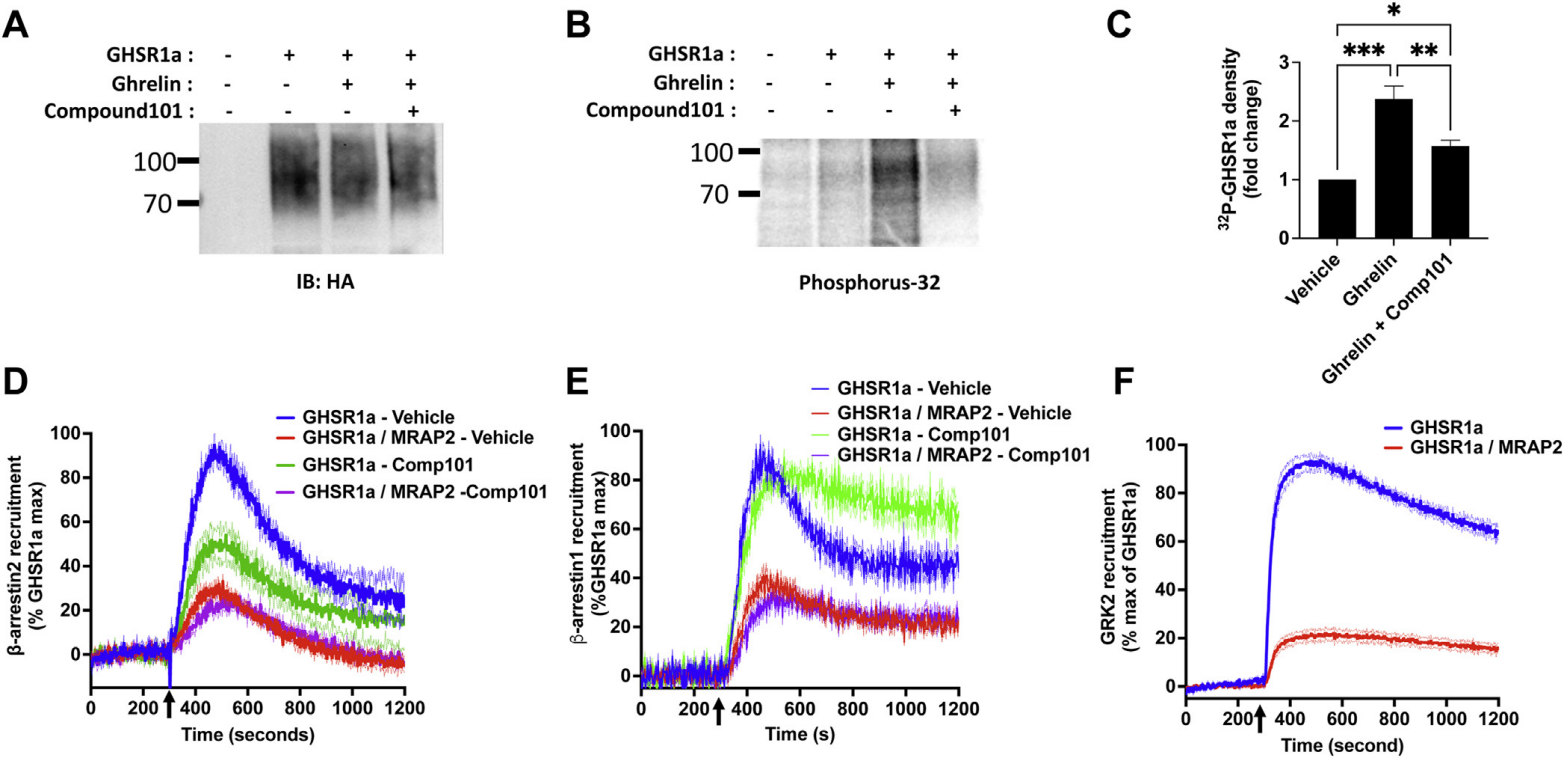
**MRAP2 inhibits GRK2 interaction with GHSR1a.***A*, Western blot detection of immunoprecipitated 3HA-GHSR1a from cells transfected with 3HA-GHSR1a, loaded with ^32^P, and treated with vehicle, ghrelin, or ghrelin and Compound 101. *B*, phosphoimaging detecting ^32^P incorporation in GHSR1a in the same samples shown in (*A*). *C*, densitometry analysis of GHSR1a phosphorylation in samples from cells treated with vehicle, ghrelin, or ghrelin and Compound 101. *D*, ghrelin stimulated β-arrestin2 recruitment to GHSR1a with and without MRAP2 in the presence or absence of Compound 101. *Arrow* indicates ghrelin injection time. *E*, ghrelin stimulated β-arrestin1 recruitment to GHSR1a with and without MRAP2 in the presence or absence of Compound 101. *F*, ghrelin stimulated GRK2DN recruitment to GHSR1a with and without MRAP2. Data are the mean ± SEM of three independent experiments. ∗ *p* < 0.05, ∗∗*p* < 0.01, ∗∗∗*p* < 0.001. GHSR1a, growth hormone secretagogue receptor 1a; HA, hemagglutinin.

GRK2 and PKC appear to be the main kinases responsible for GHSR1a phosphorylation (15). Since MRAP2 prevents the phosphorylation of GHSR1a, we hypothesized that the MRAP2–receptor interaction precludes receptor kinase access. To confirm that GRK2 phosphorylates GHSR1a, we first measured ghrelin-stimulated ^32^P incorporation in GHSR1a in the presence or absence of the GRK2/3 inhibitor Compound 101. We found that Compound 101 significantly decreased ghrelin-stimulated GHSR1a phosphorylation (Fig. 4, *A*–*C*), thus confirming that GHSR1a is a substrate of GRK2 and/or GRK3. Next, to determine if GRK2 inhibition reduces β-arrestin2 recruitment to GHSR1a, CHO cells transfected with GHSR1a-LgBiT and SmBiT-β-arrestin2 in the presence or absence of MRAP2 were treated with vehicle or Compound 101. Compound 101 treatment inhibited ghrelin-stimulated β-arrestin2 recruitment by about 50%, further confirming that GRK2-mediated phosphorylation of GHSR1a is involved in recruitment of β-arrestin (Fig. 4*D*). Again, MRAP2 inhibited β-arrestin recruitment by about 70% and the effect of MRAP2 and Compound 101 were not additive (Fig. 4*D*), suggesting that MRAP2-mediated reduction of β-arrestin recruitment to GHSR1a may involve the inhibition of multiple kinases as inhibition of GRK2 alone did not completely replicate the MRAP2 effect. We then performed the same experiment in cells transfected with GHSR1a-LgBiT and SmBiT-β-arrestin1. In contrast to β-arrestin2, pretreatment of cells with Compound 101 did not prevent β-arrestin1 recruitment to GHSR1a following ghrelin stimulation (Fig. 4*E*). This suggests that GRK2 and/or GRK3-mediated phosphorylation of GHSR1a contributes to β-arrestin2 but not β-arrestin1 recruitment.

To determine if MRAP2 affects the interaction of GRK2 and GHSR1a, we generated NanoBiT constructs to kinetically and quantitatively measure the recruitment of GRK2 to GHSR1a. Our original approach involved fusing SmBiT to the N terminus of unmodified GRK2 and cotransfect the tagged kinase with GHSR1a-LgBiT; however, this assay did not display sufficient sensitivity likely due to the transient nature of the interaction between the kinase and the receptor. Therefore, to improve the assay and obtain measurable signal, we fused SmBiT to the N terminus of the kinase dead dominant negative mutant GRK2-K220R with which the interaction with receptors is more sustained. As such, luminescence recorded from CHO cells transfected with SmBiT-GRK2DN and GHSR1a-LgBiT was readily detectable. Upon addition of ghrelin, we observed a rapid increase in GRK2 recruitment, which was significantly inhibited by coexpression of MRAP2 (Fig. 4*F*), thus demonstrating that MRAP2 interferes with the interaction of GRK2 with GHSR1a. This action of MRAP2 explains the decreased phosphorylation of the receptor and inhibition of β-arrestin2 recruitment observed.

### Essential domains for MRAP2-mediated inhibition of β-arrestin2 recruitment are also required for preventing GRK2 interaction with GHSR1a

**Figure 5:**
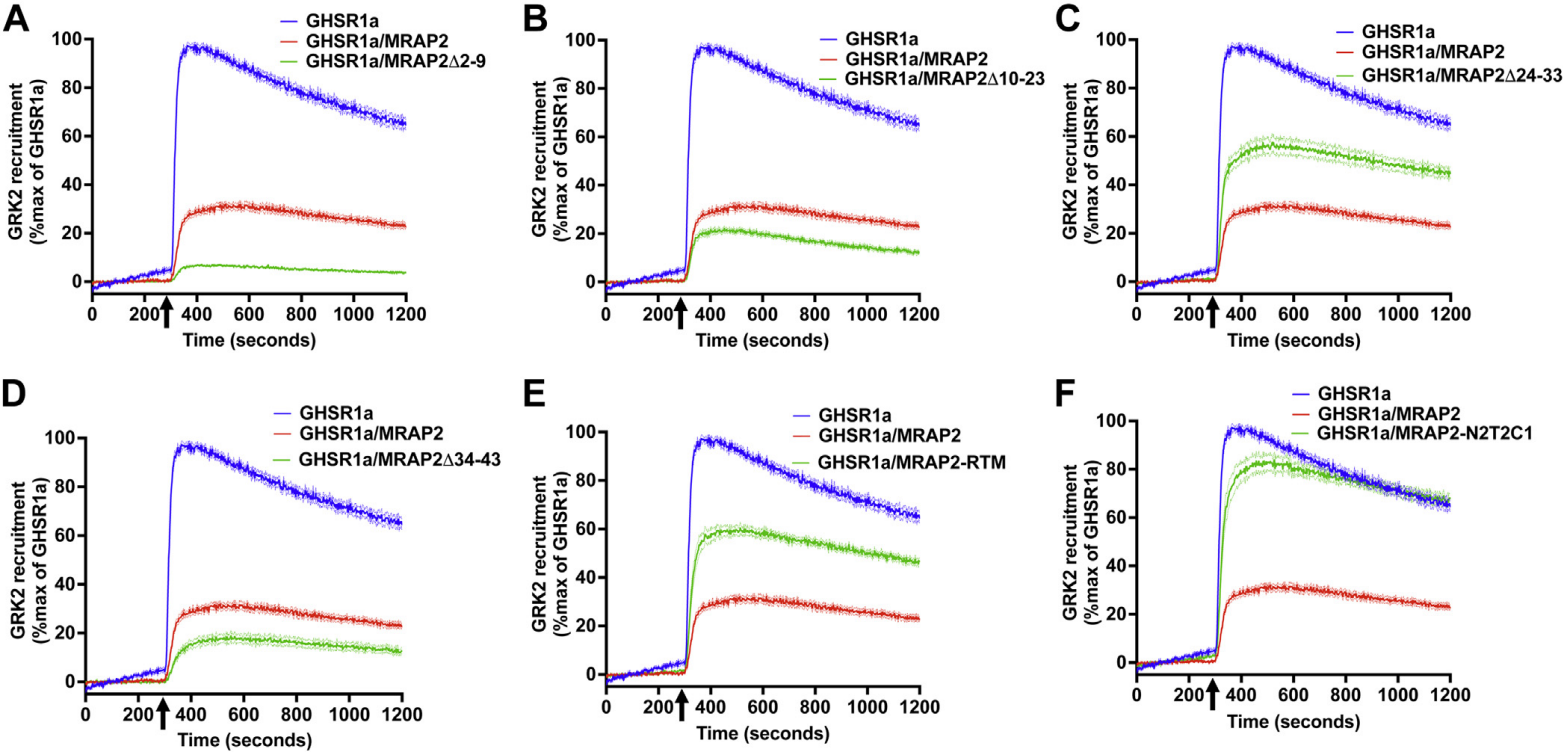
**Requirements of various regions of MRAP2 for the inhibition of GRK2 to GHSR1a.***A*–*F*, ghrelin-stimulated recruitment of GRK2DN to GHSR1a alone or coexpressed with MRAP2, MRAP2Δ-2-9 (*A*), MRAP2Δ10-23 (*B*), MRAP2Δ24-33 (*C*), MRA2Δ34-43 (*D*), MRAP2-RTM (*E*), or MRAP2N2T2C1 (*F*). Data are the mean ± SEM of three independent experiments. GHSR1a, growth hormone secretagogue receptor 1a.

We have previously shown that amino acids 24 to 33, the transmembrane domain, and the C-terminal region of MRAP2 are essential for the inhibitory function of MRAP2 on ghrelin-stimulated β-arrestin2 recruitment to GHRS1a (17). Therefore, we hypothesized that those same regions of MRAP2 are required to block the GRK2/GHSR1a interaction. Thus, we measured ghrelin-stimulated SmBiT-GRK2DN recruitment to GHSR1a-LgBiT in the absence or presence of WT MRAP2 or the following MRAP2 mutants; MRAP2Δ2-9, MRAP2Δ10-23, MRAP2Δ24-33, MRAP2Δ34-43, MRAP2-RTM, and MRAP2-N2T2C1. Consistent with our previous findings, MRAP2 mutants lacking amino acid regions 2 to 9, 10 to 23, and 34 to 43, which retain their inhibitory action on β-arrestin2 recruitment and also maintained their ability to reduce GRK2 interaction with GHSR1a (Fig. 5, *A*, *B* and *D*). In contrast, either deletion of amino acids 24 to 33 (MRAP2Δ24–33), replacement of the transmembrane domain of MRAP2 with the transmembrane domain of RAMP3 (MRAP2-RTM), or substituting the C-terminal region of MRAP2 with the C-terminal region of MRAP1 (MRAP2-N2T2C1) resulted in a significant reduction in MRAP2-mediated inhibition of GRK2 recruitment (Fig. 5, *C*, *E* and *F*). This experiment demonstrates that the regions of MRAP2 implicated in the inhibition of β-arrestin2 recruitment to GHSR1a are also important for the inhibition of GRK2 recruitment, which further implies that the inhibition of β-arrestin2 recruitment by MRAP2 is, at least in part, due to the inhibition of GRK2-mediated GHSR1a phosphorylation.

### MRAP2 inhibits PKCα recruitment to GHSR1a

**Figure 6:**
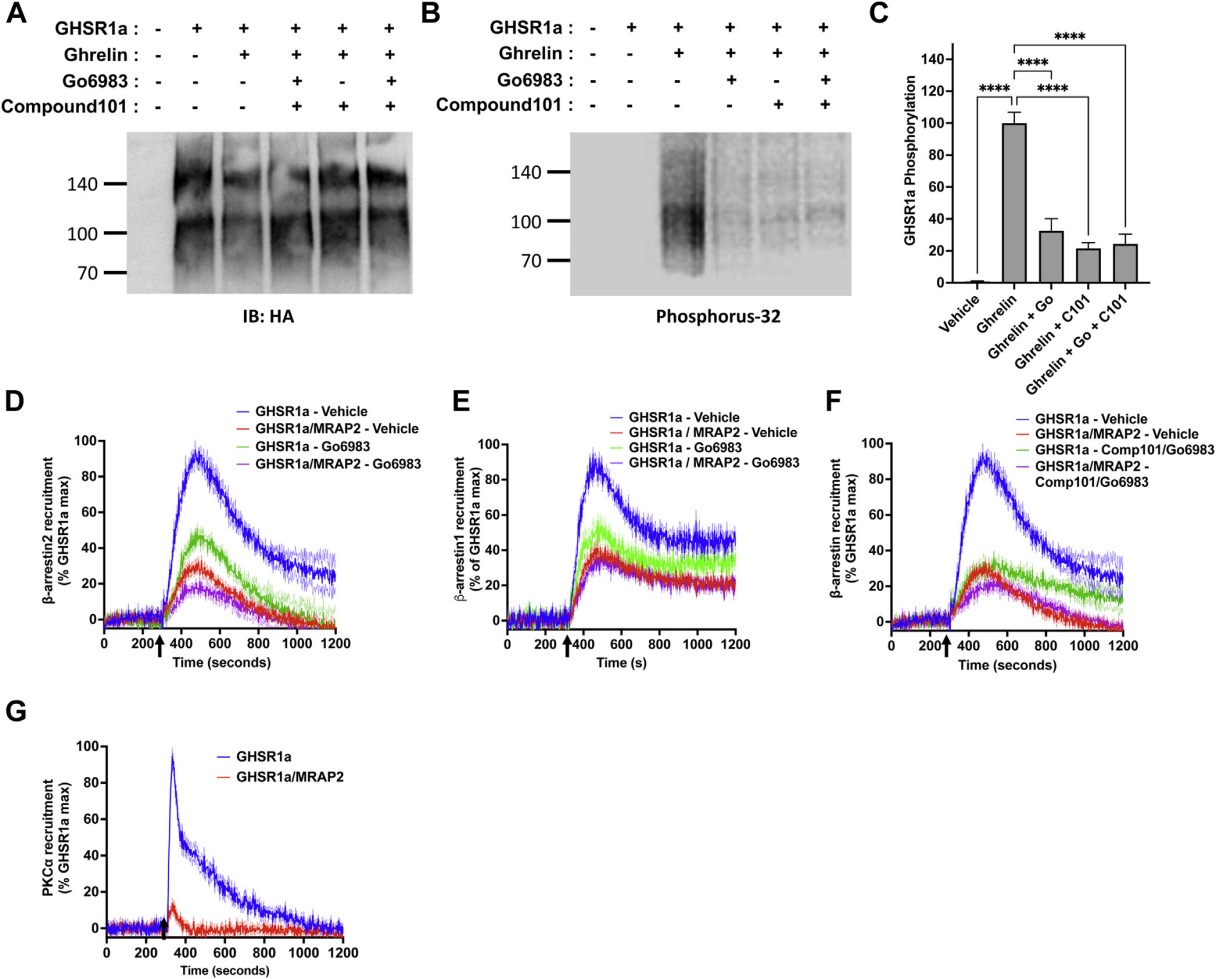
**MRAP2 inhibits PKCα recruitment to GHSR1a.***A*, Western blot detection of immunoprecipitated 3HA-GHSR1a from cells transfected with 3HA-GHSR1a, loaded with ^32^P, and treated with vehicle, ghrelin, ghrelin and Go6983, ghrelin and Compound 101, or ghrelin and both Go6983 and Compound 101. *B*, phosphoimaging detecting ^32^P incorporation in GHSR1a in the same samples shown in (*A*). *C*, densitometry analysis of GHSR1a phosphorylation in samples from cells treated with vehicle, ghrelin, ghrelin and Go6983, ghrelin and Compound 101, or ghrelin and both Go6983 and Compound 101. *D*, ghrelin stimulated β-arrestin2 recruitment to GHSR1a with and without MRAP2 in the presence or absence of Go6983. *Arrow* indicates ghrelin injection time. *E*, ghrelin stimulated β-arrestin1 recruitment to GHSR1a with and without MRAP2 in the presence or absence of Go6983. *F*, ghrelin stimulated β-arrestin2 recruitment to GHSR1a with and without MRAP2 in the presence or absence of Compound 101 and Go6983. *G*, ghrelin stimulated PKCα recruitment to GHSR1a with and without MRAP2. Data are the mean ± SEM of three independent experiments. ∗∗∗∗*p* < 0.0001. GHSR1a, growth hormone secretagogue receptor 1a; HA, hemagglutinin.

While the inhibition of GRK2 decreased β-arrestin2 recruitment to GHSR1a, it did not achieve the same inhibition as MRAP2. Additionally, the inhibitory actions of MRAP2 and Compound 101 were not additive suggesting that MRAP2 may prevent the recruitment of additional kinases to the receptor. For this reason, we tested whether PKC phosphorylates GHSR1a and participates in the initiation of β-arrestin recruitment. To determine if PKC phosphorylates GHSR1a, we transfected CHO cells with 3HA-GHSR1a, loaded the cells with 32P, and stimulated GHSR1a with ghrelin with or without the PKC inhibitor Go6983 and/or the GRK2 inhibitor Compound 101. Ghrelin caused a significant increase in ^32^P incorporation in GHSR1a, which was drastically reduced in the presence of Go6983 (Fig. 6, *A*–*C*). Compound 101 and the combination of Go6983 and Compound 101 also blocked GHSR1a phosphorylation (Fig. 6, *A*–*C*). To test the importance of PKC for ghrelin-stimulated β-arrestin recruitment, CHO cells expressing GHSR1a-LgBiT and either SmBiT-β-arrestin2 or SmBiT-β-arrestin1 with or without MRAP2 were treated with vehicle or the PKC inhibitor Go6983 before measuring ghrelin-stimulated β-arrestin recruitment. Pharmacological PKC inhibition resulted in a 50% reduction in both ghrelin-stimulated β-arrestin2 (Fig. 6*D*) and β-arrestin1 (Fig. 6*E*) recruitment, suggesting that, unlike for GRK2, GHSR1a phosphorylation by PKC is involved in the recruitment of both β-arrestin isoforms. Here again, the inhibition of β-arrestin recruitment by MRAP2 was greater than the inhibition mediated by Go6983, suggesting that inhibition of PKC alone is not sufficient to fully replicate the effect of MRAP2. Interestingly, inhibition of both GRK2 and PKC by combining Compound 101 and Go6983 resulted in further inhibition of β-arrestin2 recruitment (Fig. 6*F*), thereby mimicking the effect of MRAP2, consistent with the hypothesis that MRAP2 prevents the interaction of both GRK2 and PKC with GHSR1a. To verify that MRAP2 also blocks PKCα recruitment to GHSR1a, we generated a SmBiT-PKCα construct. Ghrelin-stimulated PKCα interaction with GHSR1a was measured in real time by transfecting CHO cells with GHSR1a-LgBiT and SmBiT-PKCα. In contrast to the assay, we developed for GRK2; recruitment of SmBiT-PKCα to GHSR1a-LgBiT was readily measurable without requiring the use of a kinase dead form of the enzyme. Whereas, ghrelin caused a rapid yet transient recruitment of PKCα to GHSR1a, expression of MRAP2 almost completely abrogated this effect (Fig. 6*G*). These results demonstrate that the inhibition of GHSR1a phosphorylation by MRAP2 is achieved by preventing GRK2 and PKCα interaction with GHSR1a.

## Discussion

GPCRs regulate numerous physiological functions by transmitting extracellular signals to intracellular domains utilizing G-protein–dependent and β-arrestin-dependent pathways. Biased ligands that preferentially activate either the G-protein or β-arrestin signaling pathway are valuable tools to study the functional consequence of each pathway separately or to promote the development of drugs with improved clinical outcomes (19, 20, 21, 22). Endogenously, however, few proteins have been shown to cause GPCR signaling bias and the molecular mechanisms involved are unclear. MRAP2 regulates multiple GPCRs (23) including the melanocortin-4 receptor (24, 25, 26), prokineticin receptors (27, 28, 29), orexin receptors (28) and the ghrelin receptor GHSR1a (17, 18). In some cases, MRAP2 plays an inhibitory role by altering receptor trafficking (27, 29) whereas in others, MRAP2 modulates signaling by increasing agonist-mediated receptor responses (14, 18, 24). Both MC4R and GHSR1a agonist-stimulated signaling are potentiated by MRAP2, and in both cases, MRAP2 lowers the constitutive activity of the receptor (18, 24). However, while a fair amount is known about the impact of MRAP2 on GPCR signaling, the molecular mechanism is poorly understood. GHSR1a can recruit both β-arrestin1 and β-arrestin2 (16). We have recently shown that MRAP2 inhibits β-arrestin recruitment to GHSR1a and associated downstream signaling (17); however, the mechanism through which MRAP2 prevents β-arrestin recruitment was still unclear.

In this study, we first investigated the importance of different phosphorylation sites within GHSR1a for its interaction with β-arrestin. While the canonical interaction of β-arrestin with GPCRs is thought to be initiated by the engagement of β-arrestin with the phosphorylated C-terminal tail of the receptor and then, in some cases, progresses with the interaction of β-arrestin with one or more intracellular loops of the receptor, several other interaction modes have been described (30, 31). We first confirmed that substitution of all the putative phosphorylation sites comprised within the C-terminal tail of GHSR1a to alanine almost completely abolished the interaction of β-arrestin with the activated receptor. However, the expression of the mutated receptor at the plasma membrane was also drastically reduced, possibly due to misfolding; it was difficult to draw conclusions regarding the importance of C-terminal tail phosphorylation in β-arrestin recruitment.

Previously, three putative phosphorylation sites within the distal C-terminal tail of GHSR1a (Ser^362^, Ser^363^, and Thr^366^) were shown to be significant for β-arrestin recruitment (16). Therefore, to further identify its role, we assessed the ability of a C-terminal tail truncated form of GHSR1a to recruit β-arrestin. In this case, GHSR1aΔct was expressed at the plasma membrane and recruitment of β-arrestin1 and β-arrestin2 was not impaired by distal C-terminal region deletion. This result suggests that, in contrast with the canonical model of β-arrestin binding to GPCRs, the interaction of β-arrestin with GHSR1a does not require attachment of β-arrestin to the phosphorylated C-terminal tail of the receptor. Since the phosphorylation of multiple sites within the C-terminal region of the intact GHSR1a is important for β-arrestin recruitment (16), but truncation of the distal portion of the C-tail containing those phosphorylation sites does not impair β-arrestin interaction with the receptor, it is possible that the unphosphorylated C-tail precludes access of β-arrestin to the ICL3. Our results suggest that this lack of access of β-arrestin to the core of the receptor could be lifted by either the phosphorylation or the truncation of the C-terminal region. Furthermore, we also demonstrated the importance of two putative phosphorylation sites within the ICL3 of GHSR1a, which when substituted to alanine resulted in a significant reduction in ghrelin-stimulated β-arrestin recruitment, further implying that interaction of β-arrestin occurs with the receptor core.

GPCR desensitization and recruitment of β-arrestin is triggered by the phosphorylation of receptors by GRKs and other kinases. Although many studies have identified how GRKs are activated and localized to GPCRs as well as how they regulate receptor activity, little is known about endogenous inhibitory mechanisms regulating GRKs themselves. Although inhibition of GRK5 by calmodulin (32, 33) and PKC (34) have been demonstrated, the role of single transmembrane GPCR accessory proteins like MRAP2 in regulating receptor phosphorylation and kinase recruitment has not previously been identified. Here, our results suggest that MRAP2 inhibits β-arrestin recruitment by preventing the interaction of kinases with the ghrelin receptor and consequently its phosphorylation. Further studies will be required to determine if this is achieved through steric hindrance, conformational alteration of the receptor, or possibly direct binding to kinases. In addition, our results also suggest that, whereas GRK2-mediated phosphorylation of GHSR1a is required for β-arrestin2 but not for β-arrestin1 recruitment, PKC promotes recruitment of both β-arrestins to the receptor.

As MRAP2 enhances the ghrelin-stimulated activation of Gα_q/11_ and decreases β-arrestin signaling, we hypothesize that, in cell types expressing both GHSR1a and MRAP2 (like AGRP neurons, pancreatic δ-cells, and pituitary somatotrophs), ghrelin preferentially stimulates Gα_q/11_-dependent signaling. However, it is also likely that in cells that express GHSR1a but not MRAP2 (like cardiomyocytes), ghrelin preferentially activates a signaling cascade downstream of β-arrestin. Ultimately, these results have potentially important implications when it comes to identifying drugs targeting GHSR1a. For example, it is possible that a β-arrestin-biased agonist of GHSR1a could provide cardioprotection without increasing appetite through activation of AGRP neurons. Finally, it is also possible that molecules that interfere with the interaction of GHSR1a with MRAP2 could reduce ghrelin action centrally to treat obesity without having deleterious cardiovascular effects.

## Experimental procedures

### Cell culture

CHO-K1 and CHO-T-REx cells were cultured in Dulbecco's modified Eagle's medium (DMEM)/F-12 (ThermoFisher) supplemented with 5% v/v of a 1/1 mix of calf bovine serum and fetal bovine serum and 1% penicillin–streptomycin. Cells were incubated at 37 °C in a humidified atmosphere containing 5% CO_2_. Cells were transfected with LipoD293 DNA Transfection Reagent from SignaGen laboratory (catalog no.: #SL100668).

### Plasmids

The 3HA-GHSR1a plasmid was obtained from the Missouri S&T cDNA Resource Center. Plasmids coding for mutants of MRAP2 and β-arrestin recruitment were previously described (17). GRK2-K220R was purchased from Addgene (plasmid #35403) and used as a template for PCR amplification and cloning in the pBiT2.2-N vector (Promega) to produce SmlBiT-GRK2DN. PKCα was cloned from HEK293 cells and inserted in pBiT2.2-N vector. To generate the bigenic GHSR1a-TetO2-MRAP2 plasmid, the HSVTK promoter was amplified from pBiT1.1-N, PCMV-TetO2 was amplified from the pcDNA5/FRT/TO vector, 3HA-GHSR1a and MRAP2 were amplified from the plasmids mentioned previously. Those four fragments were assembled using the NEBuilder Hifi DNA assembly kit (NEB). GHSR1a-S252A and GHSR1a-T261A were produced using the Q5 site-directed mutagenesis kit (NEB). To produce the GHSR1a-ΔpA mutant, the full sequence was synthetized as a gBlock (Integrated DNA Technologies) and subcloned by PCR in pBiT1.2 (C-terminal LgBiT). Plasmids were validated by sequencing.

### Cell ELISA

CHO cells were plated in 12-well plates, and triplicate wells were transfected with indicated plasmids. The next day, cells were rinsed with PBS and fixed for 10 min in 4% paraformaldehyde. Paraformaldehyde was washed three times with PBS, and cells were blocked with 5% nonfat dried milk in either PBS (nonpermeabilized) or radioimmunoprecipitation assay buffer (permeabilized) for 1 h at room temperature (RT) on a shaker. Cells were incubated with primary antibody ant-HA (mHA.11) diluted at 1/5000 in blocking buffer for 2 h at RT on a shaker. Cells were washed three times for 5 min with PBS at RT on a shaker and incubated with secondary antibody (antimouse-horseradish peroxidase, 1:5000) in blocking buffer for 1 h at RT. Cells were washed three times for 5 min with PBS before adding 200 μl of 3,3′,5,5′tetramethylbenzidine (Sigma–Aldrich). The reaction was stopped with 200 μl of 10% sulfuric acid. Three hundred liters of each sample was transferred to a 96-well plate, and absorbance was measured at 450 nm using a SpectraMax i3 plate reader.

### Inositol phosphate quantification

CHO cells were plated in 6-well plates and transfected with the indicated plasmids. Using TrypLE Express (Thermo Fisher Scientific), cells were then lifted and resuspended in 500 μl of DMEM/F12. Cell suspension (7 μl/well) was transferred in a white opaque 384-well plate. IP1 accumulation assay was performed using the IP-One kit (Cisbio) following manufacturer’s manual. Seven microliters of agonist diluted in stimulation buffer was added to each well and incubated for 1 h at 37 °C. Cells were then lysed and incubated with the detection reagents. Homogenous time-resolved fluorescence reading was performed on a SpectraMax i3 plate reader (Molecular Devices). Each condition was run in triplicate, and experiments were repeated independently at least three times.

### β-arrestin and kinase recruitment assay

CHO-K1 cells seeded in 6-well plates were transfected with HA-GHSR1a-LgBiT and either SmlBiT-β-arrestin2, SmlBiT-β-arrestin1, SmlBiT-GRK2DN, or SmlBiT-PKCa with either empty vector or MRAP2. Cells were plated in a white 384-well plate (clear bottom), Nanobit substrate was added, and the plate was read with a FLIPR Tetra automated kinetic plate reader (Molecular Devices). A separate plate with agonist was prepared and placed in the FLIPR Tetra. Luminescence was measured in every well at a sampling rate of 3 to 5 s. After 5 min of recording without agonist to allow measurement of the basal luminescence signal, agonist was injected and luminescence was measured for an additional 15 min. Baseline signal was subtracted for each well. Ghrelin was injected at a final concentration of 1 μM and when indicated, cells were preincubated with Compound 101 and/or Go6983 for 30 min before the experiment at 30 μM and 1 μM, respectively. Each condition was run in triplicate and experiments were repeated independently at least three times.

### GHSR1a phosphorylation assay and Western blot

To test the effect of MRAP2 on GHSR1a phosphorylation, CHO-T-Rex cells were seeded in 6-well plates and transfected with the bigenic GHSR1a-TetO2-MRAP2 plasmid sin the presence or absence of 1 μg/ml tetracycline. To test the effect of Compound 101 and Go6983, we used CHO cells transfected with 3HA-GHSR1a. Twenty-four hours after transfection, cells were serum starved for 4 h, washed three times with phosphate-free Krebs/Hepes buffer, and incubated with 140 μCi/well of phosphorus-32 in phosphate-free Krebs/Hepes buffer for 1 h at 37 °C. Cells were then stimulated with 1 μM ghrelin for the indicated time, placed on ice, and lysed. Lysates were centrifuged at 13,000 rpm for 10 min at 4 °C, and supernatants were incubated with anti-HA antibody 1/5000 (catalog no.: #901515; BioLegend) overnight at 4 °C before purifying immunocomplexes by incubating with Dynabeads protein G for 1 h 4 °C. Beads were resuspended in lithium dodecyl sulfate sample buffer, heated at 60 °C for 30 min, and separated by SDS-PAGE.

For Western blot, proteins were transferred on polyvinylidene difluoride membrane. Membrane was blocked with 5% nonfat dry milk in PBS with Tween 20 (PBST), incubated with primary antibody (rabbit anti-HA (C29F4, Cell Signaling) at 1/5000, and rabbit polyclonal anti-MRAP2 antibody 1/1000) in blocking buffer overnight at 4 °C. Membranes were washed three times 5 min with PBST, incubated with secondary antibody (goat anti-rabbit–horseradish peroxidase conjugate [catalog no.: #1721019; Bio-Rad]) 1/5000 in 5% milk PBST for 1 h at RT, and washed time 5 min with PBST before imaging using SuperSignal West Pico PLUS Chemiluminescent Substrate (catalog no.: # 34579) and an iBright imager.

For measurement of ^32^P incorporation, gels were dried and phosphorimaging was performed using a Typhoon FLA 9500 (GE Healthcare).

## Data availability

All data described are contained within the article.

## Conflict of interest

The authors declare that they have no conflicts of interest with the contents of this article.
